# Tetrandrine slows the radiographic progression of progressive massive fibrosis in pneumoconiosis: a retrospective cohort study

**DOI:** 10.1186/s12890-023-02577-3

**Published:** 2023-08-09

**Authors:** Meian Tang, Fu Tan, Yufei Luo, Xiongbin Xiao, Xiaobin Deng, Shenlei Li, Xiaojiang Tan

**Affiliations:** 1 Department of Respiratory and Critical Care Medicine, Nanfang Hospital, Southern Medical University, Guangzhou, China; 2 Department of Occupational Medicine, Hunan Prevention and Treatment Institute for Occupational Diseases, Changsha, China; 3 Department of Radiology, Hunan Prevention and Treatment Institute for Occupational Diseases, Changsha, China; 4 School of Biomedical Sciences, Hunan University, Changsha, China; 5 Department of Pharmacy, Hunan Prevention and Treatment Institute for Occupational Diseases, Changsha, China

**Keywords:** Tetrandrine, Progressive massive fibrosis, Pneumoconiosis, Silicosis, HRCT, Pulmonary function

## Abstract

**Objective:**

This study aims to explore the clinical effect of Tetrandrine (Tet) on progressive massive fibrosis (PMF) of pneumoconiosis.

**Methods:**

This retrospective study collected 344 pneumoconiosis patients with PMF, and 127 were eligible for the final analysis, including 57 patients in the Tet group and 70 patients in the control group. The progress of imaging and lung function were compared between the two groups.

**Results:**

After 13 months (median) of treatment, the size of PMF was smaller in the Tet group than that in the control group (1526 vs. 2306, *p*=0.001), and the size was stable in the Tet group (1568 vs. 1526, *p*= 0.381), while progressed significantly in the control group (2055 vs. 2306, *p*=0.000). The small nodule profusion and emphysema were also milder than that in the control group (6.0 vs. 7.5, *p*=0.046 and 8.0 vs. 12, *p*=0.016 respectively). Pulmonary ventilation function parameters FVC and FEV_1_ improved in the Tet group (3222 vs. 3301, *p*=0.021; 2202 vs. 2259, *p*=0.025 respectively) and decreased in the control group (3272 vs. 3185, *p*= 0.00; 2094 vs. 1981, *p*=0.00 respectively). FEV_1_/FVC was also significantly higher in the Tet group than that in the control group (68.45vs. 60.74, *p*=0.001). However, similar result was failed to observed for DLco%, which showed a significant decrease in both groups.

**Conclusion:**

Tet has shown great potential in the treatment of PMF by slowing the progression of pulmonary fibrosis and the decline of lung function.

## Introduction

Progressive massive fibrosis (PMF) is defined radiographically by the formation of large (diameter ≥ one cm) opacities which is the well-known most severe form of silicosis and coal worker’s pneumoconiosis [1]. Recent reports show that PMF is a significant and increasing problem throughout the world [2]. In the United States, a resurgence of PMF and rapidly progressive pneumoconiosis (RPP ) have occurred over the last two decades [3, 4]. In addition to traditional industries (such as mining and construction), many different studies have reported PMF in other new industries (such as denim sandblasting, artificial quartz stone exposure) [4, 5]. Undoubtedly, removal from exposure is not enough and effective treatments are urgently needed. With great regret, no clinically validated effective treatment to prevent PMF is available by recent literature [2, 6]. Tetrandrine (Tet) as a potent calcium channel blocker and in the treatment of different health issues has been referenced in *Chinese Pharmacopoeia* for its use as an anti-silicosis agent [7]. Previous studies have reported that silicotic nodules under X-ray have reduced and lung function have improved after Tet treatment [8, 9]. However, the effect of Tet on PMF has not been reported. To explore the clinical effect of Tet on PMF, we collected patient data and conducted a retrospective cohort study.

## Methods

### Study design and participants

This study was a retrospective cohort study; we analyzed the clinical data of pneumoconiosis patients with PMF who were treated in Hunan Prevention and Treatment Institute for Occupational Diseases between January 2020 to January 2022. Patients who took Tet regularly (80 mg tid, for 6 days a week, with none on the 7th day) for ≥ 6 months were defined as the Tet treatment group, and those who took Tet for < 1 month/year were defined as the control group. Patients were enrolled to this study : (1) diagnosed as coal workers’ pneumoconisis or silicosis and combined with PMF; (2) willing to participate in the study and signed an informed consent form. Exclusion criteria: (1) Tet administration was not consistent with the Tet group or the control group; (2) lack of pre - and/or post-treatment imaging data; (3) comorbidites such as tuberculous mycobacterial infection, lung tumor, respiratory infection, pneumothorax, pleural effusion, and asthma, interstitial lung diseases and significant other organ dysfunction were also excluded. Pneumoconiosis according to a national criterion on the diagnosis of occupational pneumoconiosis (GBZ 70-2015) [10], which is consistent with the criteria for pneumoconiosis of the International Labour Organization (ILO) classification [1]. Small opacities and emphysema were assessed using the International Classification of HRCT of Occupational and Environmental Respiratory Diseases [11]. PMF is defined radiographically by the formation of large (diameter ≥ 1 cm) opacities [2]. PMF size was calculated as transverse diameter × long diameter (mm) according to the HRCT mediastinal window, and if there were 2 or more, the size was equal to the sum of the individual PMF sizes. PMF progression was defined as an increase in size of more than or equal to 10%, whereas an increase in size of less than 10% was defined as stable. All images were evaluated by two radiologists who had been engaged in the diagnosis of pneumoconiosis.

### Data collection

The following data were collected: demographics, medical history (comorbidity, complication, regimen of Tet administration), detailed occupational history (including whether engaged in drilling, and the start and end dates of employment, exposure duration and so on), and smoking status, and pack-years smoked. Smoking intensity was analysed as both a categorical (0 pack-years, 1–19 pack-years and ≥ 20 pack-years). Rate of increase in PMF size was calculated as follows: post-treatment- pretreatment PMF size/baseline size *100%. We defined progression as an increase in PMF size of ≥ 10% from baseline, and stable as an increase of < 10% .

### Pulmonary function tests

All the spirometry tests data based on criteria from the American Toracic Society and European Respiratory Society criteria [12]. Trained technicians performed pulmonary function examinations using spirometry, whole body plethysmography, and single-breath diffusing capacity for carbon monoxide. We collected pulmonary function parameters including FVC, FEV_1_, FEV_1_/FVC, DLco% before and after treatment, and unreliable spirometric data were excluded.

### Statistical analysis

Continuous data were expressed as the means ± SD or median (interquartile range) and were analyzed by the Student’s t test or Mann–Whitney U test. Paired data were analyzed with the use of a paired t test or a two-sample Wilcoxon test. Frequencies and percentages were used to describe categorical data; chi-square and Fishers exact tests were used to compare these data. *P* < 0.05 was considered statistically significant. SPSS 26.0 software (IBM Inc., Chicago, Illinois, USA); GraphPad Prism V8 (GraphPad Software, La Jolla, USA) were used for statistical analysis and to make plots. We did not impute missing data.

## Result

### Demographics

**Fig. 1:**
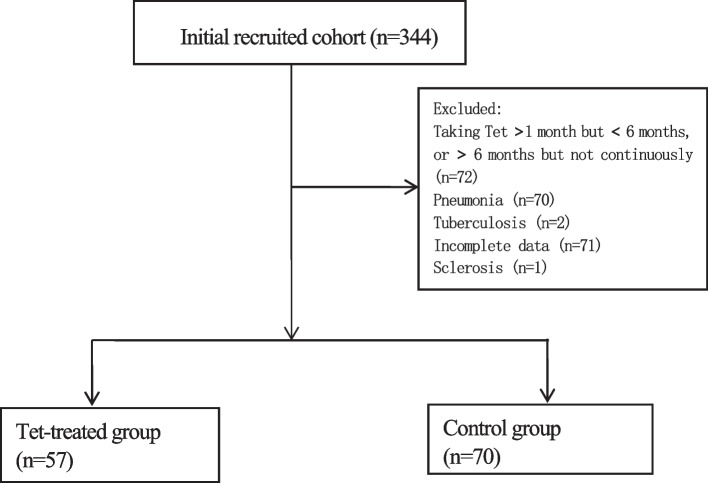
Flow of participants throughout the study

344 patients were willing to participate in this study and signed the informed consent form. 127 eligible patients participated in this study, including 57 in the Tet group and 70 in the control group (Fig. 1). The average age of the patients was 55.3 years, with a high rate of heavy smoking (smoking more than 20 pack-years) (50.4%) and a high rate of use of drill (72.4%). The average exposure duration was 13.9 years, and all patients have had ceased their exposure prior therapy.

The age of exposure were 24.9 and 42.8 years. Coal workers’ pneumoconiosis accounted for 59%, and silicosis accounted for 41%. We found there were no statistical differences in the above characteristics between the Tet and control groups at baseline. (Table 1).

**Table 1:** Demographic characteristics of the Tet and control groups

	All (*n* = 127)	Tet (*n* = 57)	Control (*n* = 70)	*P*-Value				
**Age(yrs)**	55.3 ± 7.4	55.4 ± 8.4	55.4 ± 6.6	0.970				
**Smoking status** *(pack-years)n(%)*								
* 0*	28(23.5)	15(26.8)	13(20.6)					
* 1–19*	31(26.1)	14(25.0)	17(27.0)					
* ≥ 20*	60(50.4)	27(45.0)	33(55.0)	0.732				
**Worked as a driller** *n(%)*								
* Yes*	92(72.4)	38(66.7)	54(77.8)					
* No*	34(27.6)	19(33.3)	16(22.2)	0.145				
**Exposure duration**	13.9 ± 7.6	13.6 ± 7.7	14.1 ± 7.6	0.680				
**Age at onset of dust exposure** *(yrs)*	24.8 ± 7.6	25.0 ± 7.3	24.7 ± 7.5	0.806				
**Age at the end of dust exposure** *(yrs)*	42.8 ± 8.2	42.1 ± 8.2	43.4 ± 8.2	0.403				
**Diagnosis** *n(%)*								
* Coal Worker’ pneumoconiosis*	75/127(59.1)	31(54.4)	44(62.9)					
* Silicosis*	52/127(40.9)	26(45.6)	26(37.1)	0.368				
**Interval of follow-upon** *(month) [M(P25, 75)]*	13(12, 16)	13(12, 13)	15(12, 16)	0.080				

Smoking status unavailable for 6 subjects

### The imaging characteristics(high resolution CT, HRCT)

**Fig. 2:**
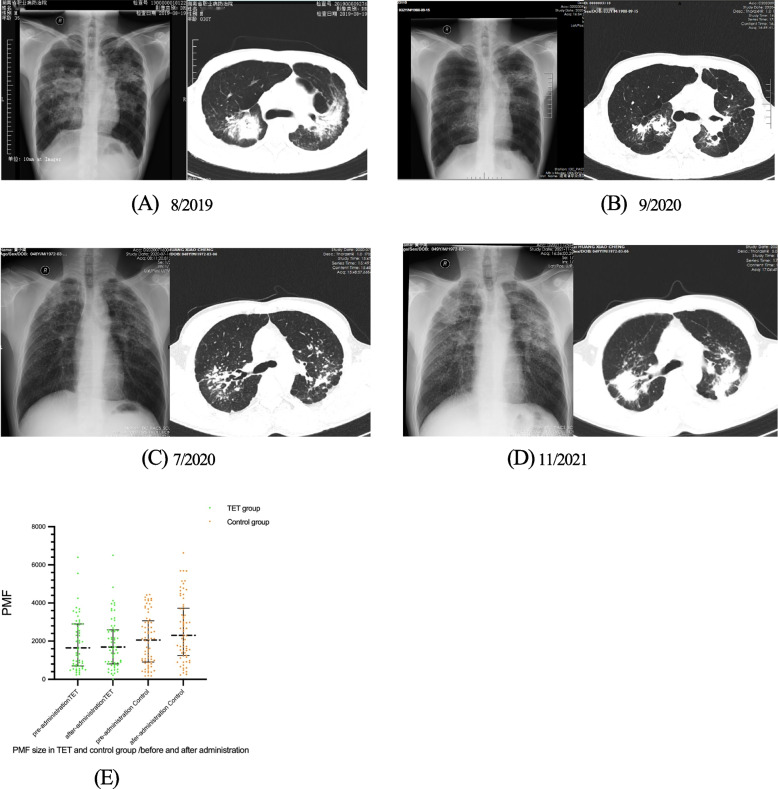
**A**,**B** This patient from the Tet group; (**A**) DR and HRCT revealed bilateral increased transparency, small opacities, multiple PMF (28×57 mm of right middle lobe, 27×35 mm of left middle lobe) and lymphadenopathies, some of which were calcific. **B** After 11 momths of Tet treatment, the PMF was significantly reduced, small opacities and multiple stripes were seen in the lungs, and bilateral increased transparency was observed. He did not receive antibiotics and anti-tuberculosis treatment, and his respiratory symptoms and lung function were also significantly improved. **C**,**D** This patient from control group, (**C)** DR And HRCT showed increased transparency, small opacities, immature PMF was seen in the right upper lung. Bilateral pleural thickening and adhesions were seen. **D** After 16 months, the PMF was significantly enlarged (25×38 mm of right upper lobe, 14×26 mm of left middle lobe). Simultaneously, the patient had worsening respiratory symptoms and pulmonary function. **E** The median PMF size decreased slightly in Tet group (1568 vs. 1526, *p*=0.21), while increased significantly in control group (2055 vs. 2306 *p*=0.00)

The imaging (HRCT) showed that there was no statistic difference in small opacity profusion and PMF size between the two groups at baseline; and after 13 months of follow-up (median), the size of PMF was smaller in the Tet group than in the control group (1526 vs. 2306, *p* = 0.001). Comparison between before and after treatment, the size of PMF was stable in the Tet group (1568 vs. 1526, *p* = 0.381), while the control group progressed significantly (2055 vs. 2306, *p* = 0.000) (Fig. 2A-E). The small opacity profusion and emphysema in the Tet group were also milder than that in the control group (6 vs. 7.5, *p* = 0.046 and 8 vs. 12, *p* = 0.016 respectively). Rate of increase in PMF size was calculated as follows: post-treatment- pretreatment PMF size/baseline size *100%. We defined progression as an increase in PMF size of ≥ 10% from baseline, and stable as an increase of < 10%. The data showed that the size of the PMF was stable in 70% of patients, progression in 30% of patients in the Tet group, while progression in 67% of patients in the control group (*p* = 0.00) (Table 2).

**Table 2:** The imaging characteristics before and after treatment between the Tet group and the control group

	Tet	Control	*P*-Value
**Samll nodule** *[M(P25, 75)]*			
* Before administration*	6.0(4.0, 8.0)	7.5(4.0, 10.0)	0.120
* After administration*	6.0(4.0, 8.0)	7.5(4.0, 10.0)	0.046
***P*** **-Value**	0.206	0.710	
**PMF size** *[M(P25, 75)]*			
* Before administration*	1568.0(682.0, 2905.0)	2055.0(904.0, 2070.0)	0.790
* After administration*	1526.0(758.0, 2528.0)	2306.0(1243.0, 3722.0)	0.001
***P*** **-Value**	0.210	0.000	
**Emphysema** *[M(P25, 75)]*			
* Before administration*	6.0 (6.0, 11.3)	8.0 (6.0, 18.0)	0.035
* After administration*	6.0 (6.0, 11.8)	12.0 (6.0, 18.0)	0.022
***P*** **-Value**	0.059	0.016	
**Rate of increase in PMF size** *[M(P25, 75)]*	-2.5(-26.3, 11.8)	21.9(5.81, 39.9)	0.001
**Patients with stable PMF** *n(%)*	40(70)	23(32.8)	
**Patients with progression PMF** *n(%)*	17(30)	47(67.2)	0.000

Progression PMF was defined as an increase in size of ≥ 10% from baseline, and stable as an increase of <10% . PMF: progressive massive fibrosis

### Spirometry parameters

**Fig. 3:**
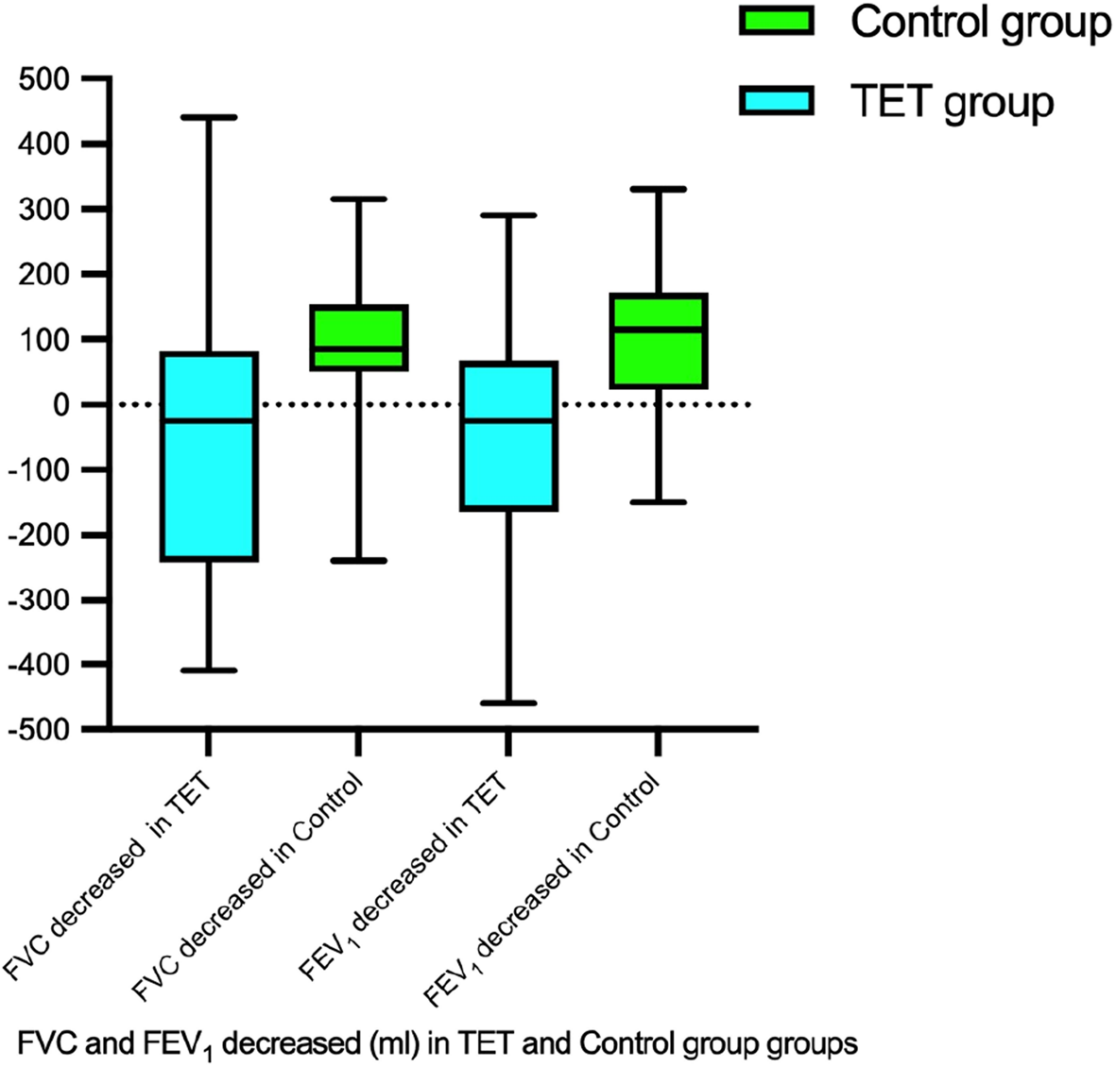
Both FVC and FEV_1_ improved by a median of 40ml in the Tet group, while they decreased by 105ml and 120ml in the control group (*p*= 0.00, *p*= 0.00 respectively)

No significant differences in FEV_1_, FVC, FEV_1_/FVC, and DLco% at baseline between the two groups was found from the data. After treatment, FVC and FEV_1_ improved in the Tet group (3222 vs. 3301, *p* = 0.021; 2202 vs. 2259, *p* = 0.025 respectively) and decreased in the control group (3272 vs. 3185, *p* = 0.00; 2094 vs. 1981, *p* = 0.00 respectively). Both FVC and FEV_1_ improved by a median of 40 ml in the Tet group, while they decreased by 105 ml and 120 ml in the control group (*p* = 0.00, *p* = 0.00, respectively ). FEV_1_/FVC in the Tet group was also significantly higher than that in the control group (68.45vs. 60.74, *p* = 0.001), with a decrease of 0.1% in the Tet group and 1.73% in the control group (*p* = 0.007) (Fig. 3). However, similar results were not observed for DLco%, which showed a statistically significant decrease in both groups (Table 3).

**Table 3:** Spirometry parameters of Tet and control group before and after treatment

		Tet	Control	*P*-Value
**FVC** *(ml)*	*Before administration*	3222 ± 650	3272 ± 652	0.690
	*After administration*	3301 ± 613	3185 ± 673	0.350
***P*** **-Value**		0.021	0.00	
**Decline in FVC** *(ml) [M(P25, 75)]*		-40(-250, 70)	85(47.5, 153.8)	0.000
**FEV**_**1**_ *(ml)*	*Before administration*	2202 ± 492	2094 ± 742	0.690
	*After administration*	2259 ± 514	1981 ± 747	0.030
***P*** **-Value**		0.025	0.000	
**Decline in FEV**_**1**_ *(ml) [M(P25, 75)]*		-40.0(-170.0, 60.0)	120.0(25.0, 172.5)	0.000
**FEV**_**1**_ **/FVC**	*Before administration*	69.33(63.68, 74.56)	64.57(54.13, 73.47)	0.450
	*After administration*	68.93(63.28, 74.95)	61.05(51.56, 71.76)	0.003
***P*** **-Value**		0.720	0.000	
**Decline in FEV**_**1**_ **/FVC** *[M(P25,75)]*		0.1(-1.39, 1.76)	1.75(-0.23, 3.45)	0.007
**DLco%**	*Before administration*	68.45 ± 14.11	70.88 ± 18.11	0.528
	*After administration*	65.50 ± 11.17	68.72 ± 16.98	0.283
***P*** **-Value**		0.000	0.003	

Twenty miners were missing spirometry parameters and were not included in the statistics presented. FEV_1_, forced expiratory volume in 1 s; FVC, forced vital capacity. DLco: difusing capacity for carbon monoxide

## Discussion

Our study investigated the efficacy of Tet in the treatment of PMF. We found a rapid increase in PMF size in control group over a short period of time, with a 21.9% (median) increase from baseline in PMF size after 15 months of follow-up. And we also found a rapid decline in lung function, with FEV_1_ falling by 120ml, FVC 85ml, FEV_1_/FVC 1.75%, and DLco% 2.3%. Recently published literature including our previous study continues to indicate that presence of PMF is associated with worsening of pulmonary function [13, 14], and indicate that development of PMF is associated with increased morbidity and mortality [15]. Hence, effective antifibrotic drugs are urgently needed to slow the progression of fibrosis and reduce the decline in lung function.

In our study, we found that the PMF size decreased by 2.5% (median) after a median 13 months of Tet treatment, while the control group increased by 21.9% (Fig. 2A-E). And 70% of PMF achieved radiographic stability, in contrast, 67.2% of the patients in the control group showed progression. Similar favorable trends were observed in emphysema and small nodule profusion after treatment. From the results of this study, we found that Tet has great potential to slow the progression of PMF fibrosis as well as the aggravation of emphysema. Similar to the results of a recent study, which showed that after taking Tet for 3–12 months, 56.5 to 65.4% of silicosis patients had improved HRCT [9].

We can also found improvement in lung function in parallel with radiographic improvement. FVC and FEV_1_ improved by a median of 40ml in the Tet group, while they decreased by 85ml and 120ml in the control group (Fig. 3). However, beneficial effect on diffusion function was failed to found; with a statistically significant decrease in both groups.

Previous studies reported that Tet combined with other drugs such as quinolyl piperazine hydroxyl.

phosphate (QOHP) or poly-2-vinyl pyridine-nitrogen oxide (PVNO) or acetylcysteine, can improve imaging, pulmonary function, and pneumoconiosis symptoms in the treatment of pneumoconiosis [16–19]. This retrospective cohort study further indicates that Tet has a potential therapeutic effect on PMF by delaying the progression of PMF and halting the rapid decline in lung function.

For decades, many studies have reported that Tet can inhibit the progression of pneumoconiosis fibrosis though different pathways. Early results showed that Tet exhibited cytoskeletal depolymerization activity by interrupting the process of collagen biosynthesis [20, 21] and degrading the collagen in the silicic nodules formed and inhibiting the transcription of collagen genes [22, 23]. Another report suggested that Tet could promote the activity of superoxide dismutase (SOD) in lung tissue [7]. Recent findings showed that Tet down-regulated the silica-induced secretion of cytokines by NLRP3 inflammasome activation [24, 25]. Song MY, et al. using multiple methods and multi-omics techniques, further confirmed that Tet can inhibit silicosis-associated inflammation and fibrosis by suppressing both the canonical and noncanonical NLRP3 inflammation pathways in lung macrophages [14].

Our data provide new evidence of Tet in the treatment of PMF which may promote an acceptable individualized treatment regime of Tet. This study has some limitations. First, this study is a retrospective study, recall bias and selective bias cannot be excluded; second, it is a single center data and the follow-up period was short. Hence, multicenter randomized controlled trial (RCT) or prospective cohort studies are expected in the near future.

## Conclusion

Tet has great potential in the treatment of PMF by slowing the progression of pulmonary fibrosis and decline of lung function.

## Data Availability

All data generated or analysed during this study are included in this published article.

## Abbreviations

FEV_1_: Forced expiratory volume in 1 s
FVC: Forced vital capacity
DLco: Difusing capacity for carbon monoxide
PMF: Progressive massive fibrosis
